# Longitudinal Study of CSF Biomarkers in Patients with Alzheimer's Disease

**DOI:** 10.1371/journal.pone.0006294

**Published:** 2009-07-17

**Authors:** Peder Buchhave, Kaj Blennow, Henrik Zetterberg, Erik Stomrud, Elisabet Londos, Niels Andreasen, Lennart Minthon, Oskar Hansson

**Affiliations:** 1 Clinical Memory Research Unit, Department of Clinical Sciences Malmö, Lund University, Lund, Sweden; 2 Neuropsychiatric Clinic, Malmö University Hospital, Malmö, Sweden; 3 Institute of Neuroscience and Physiology, Department of Neurochemistry and Psychiatry, Sahlgrenska University Hospital, Göteborg University, Göteborg, Sweden; 4 Institute of Biomedicine, Department of Clinical Chemistry and Transfusion Medicine, Sahlgrenska University Hospital, Göteborg University, Göteborg, Sweden; 5 Karolinska Institutet, Department of Neurobiology, Caring Sciences and Society, Stockholm, Sweden; VU University Medical Center and Center for Neurogenomics and Cognitive Research – VU University, the Netherlands

## Abstract

**Background:**

The CSF biomarkers tau and Aβ42 can identify patients with AD, even during the preclinical stages. However, previous studies on longitudinal changes of tau and Aβ42 in individual patients with AD and elderly controls report somewhat inconsistent results.

**Methodology/Principal Findings:**

We investigated the levels of tau and Aβ42 at baseline and after 1 year in 100 patients with AD. In a second cohort of 45 AD patients we measured the CSF biomarkers at baseline and after 2 years. Moreover, in 34 healthy elderly controls the CSF biomarkers were followed for 4 years. The baseline levels of tau were increased with >60% in AD patients compared to controls (p<0.001), while baseline Aβ42 levels were decreased with >50% (p<0.001). In the AD group followed for 2 years, tau increased with 16% compared to the baseline levels (p<0.05). However, the levels of tau were stable over 4 years in the controls. The levels of Aβ42 did not change significantly over time in any of the groups. In the patients with AD, tau was moderately associated with worse cognitive performance already at baseline (p<0.05).

**Conclusions/Significance:**

Tau and Aβ42 in CSF seem to reflect the underlying disease state in both early and late stages of AD. The slight increase in tau over time observed in the patients with AD is modest when compared to the relatively large difference in absolute tau levels between AD patients and controls. Therefore, these markers maintain their usefulness as state markers over time and might serve as surrogate markers for treatment efficacy in clinical trials.

## Introduction

Dementia is a growing health-economic issue worldwide. Alzheimer's disease (AD) accounts for most cases of dementia. The onset of AD is insidious. The molecular pathology probably starts 10–20 years before that patients with AD develop any symptoms. The major pathological features in the brains of AD patients are senile plaques, containing β-amyloid (Aβ), and neurofibrillary tangles with tau protein[1].

As novel therapies with possible disease-modifying effects are under development, there is an urgent need for surrogate markers to measure the potential biochemical effects of such therapies. Cerebrospinal fluid (CSF) tau and Aβ42 reflect the underlying disease state and might serve as such surrogate markers[2]. However, for this purpose, the intra-individual variation of the biomarker levels over time needs to be relatively small in order to identify treatment effects.

CSF biomarkers might also be used in the diagnostic work up of symptomatic AD patients, since the levels of tau are typically increased, while the levels of Aβ42 are decreased[2]. These biomarkers can also predict progression to AD in subjects suffering from mild cognitive impairment (MCI) with relatively high accuracy[3], [4], [5]. There is evidence of changes of CSF tau and Aβ42 in the even earlier, pre-symptomatic phase of the AD[6], [7]. In conclusion, the CSF levels of tau and Aβ42 seem to be substantially altered very early in the disease process of AD. Numerous studies show that in groups of AD patients there is no strong correlation between the severity of the disease stage and the levels of tau and Aβ42, indicating that the levels of the CSF biomarkers do not change substantially over time in symptomatic AD patients[2]. However, previous longitudinal studies of the levels of tau in patients with AD show somewhat conflicting results[8], [9], [10], [11], [12], [13], [14], [15]. Some studies have shown that the levels of CSF tau are stable over time[9], [10], [12], while others have shown that tau levels are increased at follow-up[8], [13]. The results of studies on the stability of Aβ42 in AD have also been inconsistent - some studies have found no change over time[10], [11], while others have found longitudinal increments[8] or decrements[14]. Because of the previous inconsistent results we conducted a longitudinal study in two rather large cohorts of patients with AD. Lumbar puncture was performed at baseline and after 1 year in 100 AD patients, while lumbar puncture was performed at baseline and after 2 year in 45 AD patients. Moreover, 34 healthy elderly controls underwent lumbar puncture at baseline and after 4 years.

## Methods

### Subjects

The study sample was recruited at the memory disorder clinic, Malmö University Hospital, Sweden. At baseline, physicians specialized in cognitive disorders performed a thorough physical, neurological and psychiatric examination, as well as a clinical interview, of each patient. Computed tomography (CT) or magnetic resonance imaging (MRI) of the brain was done, as well as routine blood analyses and analysis of *APOE* genotype. The patients were also evaluated with cognitive tests, including Mini-mental State Examination (MMSE).

The patients with AD fulfilled the DSM-IIIR criteria of dementia[16] and the criteria of probable AD defined by NINCDS-ADRDA[17]. Their cognitive symptoms could not be explained by other pathology.

The recruited 119 AD patients had undergone lumbar puncture two times with an interval of 6–30 months. In 100 of these patients, the time from baseline to the second lumbar puncture was approximately one year (6–18 months) and they are subsequently called the one-year AD cohort. In 45 AD patients the interval between baseline and follow-up lumbar puncture was two years (18–30 months) and they are subsequently called the two-year AD cohort 2. In 26 of the AD patients there were follow-up lumbar punctures performed at both one and two years and they are included in both of the cohorts. All patients were during the follow-up period continuously tested with cognitive tests including MMSE.

For the age-matched control population the inclusion criteria were: (i) absence of memory complaints or any other cognitive symptoms, (ii) preservation of general cognitive functioning, and (iii) no active neurological or psychiatric disease. At baseline, 36 controls with MMSE scores over 27 were included. The controls were followed clinically and evaluated with MMSE after 4 years, in order to rule out development of cognitive decline. At follow-up, two patients exhibited significant cognitive dysfunction and were therefore excluded. The remaining 34 controls had MMSE scores ≥27 at follow- up. Lumbar puncture was performed at baseline and after 4 years (range 3.7–4.2).

### Ethics Statement

All patients gave written informed consent to participate in the study. The study was conducted according to the provisions of the Helsinki Declaration and approved by the ethics committee of Lund University, Sweden.

### Analyses of levels of tau and Aβ42

The CSF samples were collected by lumbar puncture. The first 12 mL of CSF was collected in a polypropylene tube, directly transported to the local laboratory for centrifugation at 2000×*g* at +4°C for 10 min. The supernatant was pipetted off, gently mixed to avoid potential gradient effects, and aliquoted in 1 mL portions in polypropylene tubes that were stored at −80°C pending biochemical analyses, without being thawed and re-frozen.

In the AD patients, CSF total tau (T-tau) concentration was determined using a sandwich ELISA (Innotest hTAU-Ag, Innogenetics, Gent, Belgium) specifically constructed to measure all tau isoforms irrespectively of phosphorylation status, as previously described[18]. CSF Aβ1-42 levels were determined using a sandwich ELISA (Innotest® ß- amyloid (1–42), Innogenetics, Gent, Belgium), specifically constructed to measure Aβ containing both the first and 42^nd^ amino acid, as previously described[11]. In the 1-year AD cohort repeated measurement of CSF tau and CSF Aβ42 were done in 100 and 97 subjects, respectively. In the 2-year AD cohort repeated measurement of CSF tau and Aβ42 were done in 45 and 42 subjects, respectively.

In the cognitively normal controls, the concentrations of tau and Aβ_42_ in CSF was determined with xMAP technology using the INNO-BIA AlzBio3 kit (Innogenetics, Ghent, Belgium)[4]. Results from the Luminex xMAP system were converted to ELISA levels based on previously published conversion factors[19].

### Statistical analyses

To compare continuous baseline data between the cohorts, non-parametric Kruskal-Wallis ANOVA was performed, followed by Mann-Whitney *U*-test. Pearson's *x* ^2^ test was used for dichotomous variables. Wilcoxon signed-rank test was used to compare baseline and follow-up data in the cohorts. Spearman's correlation coefficient was used for correlation analyses. The statistical analyses were accomplished with SPSS for Windows, version 16.0.

## Results

### Baseline CSF levels of tau and Aβ42

The baseline demographic variables of the two AD cohorts and the age-matched controls are given in table 1. Both the baseline CSF levels of tau in the 1-year AD cohort (693±301 ng/l) and in the 2-year AD cohort (663±308 ng/l) were significantly elevated with 61–68%, when compared to the baseline levels in the control group (412±232 ng/l; P<0.001) (table 2). In contrast, the baseline CSF levels of Aβ42 in the 1-year AD cohort (275±103 ng/l) and in the 2-year AD cohort (288±103 ng/l) were significantly reduced with 56–58%, when compared to the baseline levels in the control group (659±179 ng/l; P<0.001) (table 2). Furthermore, the above described alterations of tau and Aβ42 were highly significant both in AD patients with low and high scores on MMSE at baseline, when compared to the healthy controls (data not shown).

**Table 1:** Demographic characteristics of the AD patients and the age-matched controls at baseline and follow-up.

	Controls, n = 34	1-year AD cohort, n = 100	2-year AD cohort, n = 45
Age, years	72±8.3	74±7.2	73±7.1
Sex, M/F	10/24	36/64	22/23
APOE ε4 carrier, %	26.5	67.7^a^	75.6^a^
Follow-up time, y	3.8±0.1	1.2±0.2	2.1±0.2
MMSE, baseline (0–30 p)	29.3±1.0	21.8±4.8^a^	23.4±4.4^a^
MMSE, follow-up (0–30 p)	28.7±1.2	20.4±5.6	20.2±6.9

Values are means±SD, except as noted otherwise.

a p<0.001 vs Controls.

Abbreviations: 1-year AD cohort, AD patients with approximately one year between baseline and follow-up lumbar puncture; 2-year AD cohort, AD patients with approximately two years between baseline and follow-up lumbar puncture; Controls, healthy controls followed for 4 years; APOE ε4 carrier, at least one apolipoprotein E ε4 allele; MMSE, Mini-Mental State Examination.

**Table 2:** Baseline and follow-up levels of CSF tau and Aβ42 in the AD patients and age-matched controls.

	Controls, n = 34	1-year AD cohort, n = 100	2-year AD cohort, n = 45
Baseline Tau, ng/L	412±232	693±301^a^	663±308^a^
Follow-up Tau, ng/L	399±194	731±402	768±528^b^
Longitudinal difference, Tau, %	−3.2	+5.5	+15.8
Baseline Aβ42, ng/L	659±179	275±103^a^	288±103^a^
Follow-up Aβ42, ng/L	651±168	296±132	321±137
Longitudinal difference, Aβ42, %	−1.2	+7.6	+11.5

Values are means±SD except if noted otherwise.

a p<0.001 vs Controls.

b p<0.05 vs baseline.

Abbreviations: CSF, cerebrospinal fluid; 1-year AD cohort, subjects with Alzheimer's disease with an interval between baseline and follow-up lumbar puncture of approximately one year; 2-year AD cohort, subjects with Alzheimer's disease with an interval between baseline and follow-up lumbar puncture of approximately two years. Controls, healthy controls followed for 4 years.

### Longitudinal changes of the CSF levels of tau and Aβ42 in the AD patients

**Figure 1:**
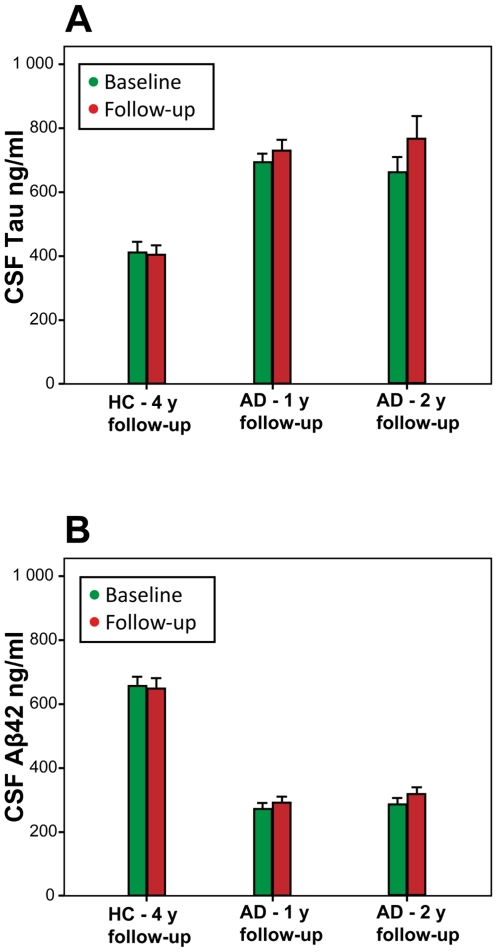
Longitudinal change of CSF tau and Aβ42. The figure shows the mean baseline and follow-up levels of CSF tau (panel A) and CSF Aβ42 (panel B) in healthy controls (HC), that were followed for 4 years (y), and in two cohorts of patients with Alzheimer's disease (AD) that were followed for one or two years, respectively. Error bars represent standard errors of the mean. The figure illustrates that differences between controls and AD patients by far surpass the within-group differences over time.

The levels of CSF tau were non-significantly higher at follow-up compared to the baseline levels in the 1-year AD cohort (P>0.05; table 2, figure 1). Also the levels of CSF Aβ42 were non-significantly higher at follow-up in this group (P>0.05; table 2, figure 1). In the 2-year AD cohort, the levels of CSF tau were significantly increased with 15.8% at follow-up, when compared to the baseline levels (p = 0.03; table 2, figure 1). Furthermore, the CSF levels of Aβ42 were non-significantly increased at follow-up compared to baseline in this group (P>0.05; table 2, figure 1).

### Longitudinal changes of the CSF levels of tau and Aβ42 in the controls

At follow-up, the CSF levels of tau were slightly and non-significantly lower compared to the baseline levels (table 2, figure 1). A modest and non-significant decrease of Aβ42 was also observed during follow-up (table 2, figure 1).

### Associations between the levels of tau and Aβ42 in CSF and cognitive performance

In the 1-year AD cohort, high levels of CSF tau at baseline, but not Aβ42, correlated moderately to poor baseline performance on the cognitive test MMSE (*r*_s_ = −0.22, p<0.05). In the 2-year AD cohort, similar correlations between the baseline levels of tau, but not Aβ42, and MMSE performance (*r*_s_ = −0.40, p<0.01) were found. However, baseline levels of tau or Aβ42 did not correlate with cognitive deterioration over time as measured with repeated MMSE during the clinical follow-up (p>0.05). Moreover, changes in tau or Aβ42 levels over time did not correlate with the rate of cognitive deterioration (p>0.05).

## Discussion

We found that the baseline levels of CSF tau were increased with around 60% in AD patients compared to controls, while baseline CSF Aβ42 levels were decreased with more than 50%. In AD patients, tau increased with 16% over two years and CSF tau was moderately associated with worse cognitive performance already at baseline. However, the levels of tau were stable over 4 years in the controls. The levels of Aβ42 did not change significantly over time in any of the groups and did not correlate with baseline cognitive performance.

Biomarkers for neurodegenerative disorders can be divided into markers of disease state and markers of disease stage or rate[20]. Markers of disease state, usually called diagnostic biomarkers, facilitate detection of a certain biological disease in populations of individuals with similar symptoms (e.g. memory impairment) caused by different conditions. A disease state marker should ideally exhibit a high diagnostic accuracy over the entire course of the disease process. Both CSF tau and Aβ42 fulfill the requirements for disease state markers of AD, because these biomarkers exhibit reasonably high specificity and sensitivity for both early and late stages of AD[2]. The results of the present study suggest that CSF Aβ42 is not a marker of disease stage or rate, since the levels are stable over time in individual patients with AD and do not correlate with cognitive function. Similarly, previous studies show that the amyloid load in the brain of AD patients, as assessed with repeated positron emission tomography (PIB-PET) measurements, keeps stable over time despite cognitive decline[21]. It might be that Aβ42 levels in both the CSF and the brain are altered during the very early preclinical stages of AD and are thereafter relatively stable during the symptomatic course of the disease.

The present study suggests that tau levels in CSF increase slightly over time. In agreement, Stefani and collaborators have found that CSF tau levels are associated with the disease stage of AD[22], a finding supported by the moderate correlation between cognitive performance and CSF tau found in the present study. Therefore, it might be that CSF tau, in addition to being a robust disease state marker, to some degree reflects the disease stage. These observations are supported by neuropathological studies showing that tau-containing neurofibrillary tangles, but not amyloid plaques, are associated with the cognitive function of AD patients[23]. However, other methods such as cognitive tests or measures of brain atrophy or cerebral blood flow are likely to prove to be more valuable as disease stage markers of AD[24], [25], [26], and CSF biomarkers should primarily be used as diagnostic markers detecting the underlying disease state[2]. Therefore, different methods should be combined when defining the disease state and stage of individual patients with cognitive dysfunction.

CSF biomarkers, such as tau and Aβ42, could possibly also be used as *surrogate markers* in clinical therapeutic AD trials. Surrogate biomarkers should be involved in the early pathophysiologic cascade and they ought to inform on biological interactions with the molecular target of the drug in humans [20]. Future disease-modifying therapies against AD may halt the degenerative process, but are not expected to have direct symptomatic effects. As a result no short-term cognitive improvements are expected in such trials. Therefore, large patient populations and extensive treatment periods will be required to identify treatment effects on clinical parameters. Data from smaller pilot investigations, using biomarkers as endpoint to determine whether a certain drug is reaching and acting on its biological target in patients with AD, would be very valuable when making a go/no-go decision for an expensive clinical trial with clinical improvement as the endpoint[2]. However, the markers used as surrogate markers in such trials must have a low intra-individual variation over time. The present study confirms previous investigations that the changes of CSF tau and Aβ42 is very modest over time in individual patients with AD[8], [9], [10], [11], [12], [13], [14], which might indicate that these CSF biomarkers may serve as sensitive tools to identify and monitor even minor biochemical changes induced by treatments that are directed against these targets, such as Aβ immunotherapy. For example, recently Lannfelt and colleagues found that PBT2 (a metal-protein attenuating compound) reduced the levels of Aβ42 in CSF, but not plasma, of patients with AD, indicating that the drug had a central effect on Aβ metabolism[27].

In conclusion, CSF tau and Aβ42 seem to reflect the underlying disease state in both early and late stages of AD. The slight increase in tau over time observed in patients with AD is modest when compared to the relatively large difference in absolute tau levels observed between AD patients and controls, indicating that tau and Aβ42 do not primarily reflect the progression of the disease over time. Therefore, these markers might serve as surrogate markers for treatment efficacy in clinical trials.
